# Inhibitory effects and mechanism of dihydroberberine on hERG channels expressed in HEK293 cells

**DOI:** 10.1371/journal.pone.0181823

**Published:** 2017-08-01

**Authors:** Dahai Yu, Lin Lv, Li Fang, Bo Zhang, Junnan Wang, Ge Zhan, Lei Zhao, Xin Zhao, Baoxin Li

**Affiliations:** Department of Pharmacology, College of Pharmacy, Harbin Medical University, Harbin, Heilongjiang Province, People’s Republic of China; University of Hull, UNITED KINGDOM

## Abstract

The human ether-a-go-go-related gene (hERG) potassium channel conducts rapid delayed rectifier potassium currents (*I*_Kr_) and contributes to phase III cardiac action potential repolarization. Drugs inhibit hERG channels by binding to aromatic residues in hERG helixes. Berberine (BBR) has multiple actions, and its hydrogenated derivative dihydroberberine (DHB) is a potential candidate for developing new drugs. Previous studies have demonstrated that BBR blocks hERG channels and prolongs action potential duration (APD). Our present study aimed to investigate the effects and mechanism of DHB on hERG channels. Protein expression and the hERG current were analyzed using western blotting and patch-clamp, respectively. DHB inhibited the hERG current concentration-dependently after instantaneous perfusion, accelerated channel inactivation by directly binding tyrosine (Tyr652) and phenylalanine (Phe656), and decreased mature (155-kDa) and simultaneously increased immature (135-kDa) hERG expression, respectively. This suggests disruption of forward trafficking of hERG channels. Besides, DHB remarkably reduced heat shock protein 90 (Hsp90) expression and its interaction with hERG, indicating that DHB disrupted hERG trafficking by impairing channel folding. Meanwhie, DHB enhanced the expression of cleaved activating transcription factor-6 (ATF-6), a biomarker of unfolded protein response (UPR). Expression of calnexin and calreticulin, chaperones activated by ATF-6 to facilitate channel folding, were also increased, which indicating UPR activation. Additionally, the degradation rate of mature 155-kDa hERG increased following DHB exposure. In conclusion, we demonstrated that DHB acutely blocked hERG channels by binding the aromatic Tyr652 and Phe656. DHB may decrease hERG plasma membrane expression through two pathways involving disruption of forward trafficking of immature hERG channels and enhanced degradation of mature hERG channels. Furthermore, forward trafficking was disrupted by impaired channel folding associated with altered interactions between hERG proteins and chaperones. Finally, trafficking inhibition activated UPR, and mature hERG channel degradation was increased by DHB.

## Introduction

The human ether-a-go-go-related gene (hERG) potassium channel conducts the rapid delayed rectifier potassium current (*I*_Kr_), thereby contributing to phase III repolarization of the cardiac action potential [1]. Functional impairment of the hERG channel can result in inherited and acquired long QT syndrome type 2 (LQT2), caused by mutations of the *hERG* or off-target effects of diverse therapeutic agents, respectively [2]. Small molecules can remarkably inhibit hERG channels by binding to a few aromatic side chains in helixes of the α subunits of the channels, which could lead to deleterious consequences such as torsade de pointes and even sudden cardiac death [3]. Hence, the hERG channel has been identified as a critical target for the detection of cardiotoxicities.

Recently, increasing attention has been focused on the cardiac safety evaluation of new drugs [4]. A multitude of drugs have been withdrawn from the market or strictly restricted in clinical applications owing to their severe adverse reactions in the cardiovascular system, although they had been approved for their significant efficacies [5]. Additionally, current paradigms of cardiac safety assessment consider hERG channel inhibition a potential risk concern, and new drug approval departments regularly require developers to provide data on the effects of drugs on hERG channel before they are marketed [6].

Berberine (BBR), an isoquinoline alkaloid extracted from the roots and spears of the *Berberis* and *Coptis* genera, has multiple pharmacological actions including cholesterol-, glucose-, and lipid-lowering in combination with statins [7–9]. Dihydroberberine (DHB) is a hydrogenated derivative of BBR [10]. Pharmacokinetic analyses have indicated that compared with BBR, DHB displayed improved absorption and enhanced oral bioavailability. In addition, it had been demonstrated that the in vivo efficacy of DHB is superior to that of BBR. Based on its pronounced effects on adiposity and improvement of glucose tolerance, DHB is currently under investigation as a drug candidate for the treatment of type 2 diabetes [11].

Furthermore, interest in the investigation of DHB has increased, and plenty of studies suggest that it has greater anti-inflammatory, anti-atherosclerotic, and hypolipidemic activities than BBR does [12]. DHB also has therapeutic potential for myotonic dystrophy type I because of its central nervous system (CNS) effects [13]. Therefore, DHB is considered a potential candidate agent for the development of new drugs and would likely be even more promising than BBR is. It has been confirmed that BBR could block the hERG channel and prolong action potential duration (APD) [14, 15]. Importantly, the combination of BBR with antibiotics enhances the inhibitory effects on the hERG channel [16]. Thus, evaluating the cardiotoxicity of DHB based on its effects on the hERG channel could facilitate the exploration of its safe and rational application.

Since previous studies have demonstrated that BBR inhibits both hERG current and the expression of hERG protein [14, 15], in this study, we aimed to investigate the potential inhibition of the current and protein of the hERG channel by DHB in HEK293 cells stably expressing the wild-type (WT) hERG. Moreover, HEK293 cells transiently transfected with hERG cDNA was used to determine the sites of hERG channel that were bound by DHB. Further, we attempted to elucidate the potential mechanisms underlying the effects of DHB on hERG channel.

## Materials and methods

### Reagents

DHB was purchased from Chroma-Biotechnology Co., Ltd. (Chengdu, China). Dimethyl sulfoxide (DMSO) was purchased from Sigma-Aldrich Corporation (USA). In order to obtain one 1mM original fluid, we dissolved DHB in 10% DMSO and 90% deionized water. Drug solution was diluted in nutrient solution before using. In the cellular experiments, cells were treated with DHB immediately or were incubated with different concentrations of DHB for 24 h. For the control group, cells were treated with DMSO with the final concentration of 1%.

### Cell culture and treatment

Human embryonic kidney 293 (HEK293) cells (purchased from Chinese Peking Union Medical College, Peking, China) were cultured in Dulbecco’s modified Eagle’s medium (DMEM, Hyclone, Logan, UT, USA) with 10% (v/v) fetal bovine serum (FBS, Gibco) at 37°C and exposed to an atmosphere of 5% CO_2_. The HEK293 cells stably expressing the WT hERG (hERG-HEK293) is a kind gift from Professor Zhiguo Wang, Harbin Medical University, P. R. China. The culture medium was also supplemented with 400 μg·mL^-1^ gentamycin (G418, Calbiochem, USA). Cells were treated with acute perfusion of DHB for detection of hERG current and hERG channel kinetics, or incubation with DHB for different time points for detection of hERG current, hERG channel kinetics, proteins of interest accordingly.

### Patch-clamp recording techniques

We used a whole-cell patch clamp technique for the measurement of hERG currents. Briefly, the cells were digested and suspended in the bath solution (136 mM sodium chloride [NaCl], 5.4 mM potassium chloride [KCl], 5 mM HEPES, 1 mM magnesium chloride [MgCl_2_], 1 mM calcium chloride [CaCl_2_], and 10 mM glucose, pH 7.4) at 4°C. The cell suspension was then transferred into a small cell bath on an inverted microscope (Olympus IX-70, Olympus Corp., Tokyo, Japan), the cells were allowed to attach to the glass bottom for about 10 min, and then they were superfused with the bath solution at a rate of 1.0 mL·min^-1^. The whole-cell configuration was maintained at a temperature of 25°C using a glass pipette with a tip resistance of 1–3 MΩ filled with pipette solution (130 mM KCl, 1 mM MgCl_2_, 5 mM ethylene glycol tetraacetic acid (EGTA), 5 mM Mg-ATP, 0.1 mM GTP and 10 mM HEPES, pH 7.3). An Axonpatch-200B amplifier (Axon Instruments Inc., Union City, CA, USA) was used to record the currents. We used a pClamp 9.2 (Axon Instruments) to control the program. The inhibition ratio of the DHB voltage-dependent peak tail current was calculated using the function (I_Control_—I_DHB_) / I_Control_. We used six concentrations of DHB (1, 5, 10, 30, 50, and 100 μM) to construct the half-maximal inhibitory concentration (IC_50_) curve, which was sigmoidal in shape plotted against the log [drug concentration] fitted with the Hill equation. The maximum % of DMSO in final experiment test solutions was 1%. hERG currents were evoked through a 4-s repolarizing step to -50 mV, following a 2-s depolarization step with a 10 mV stepwise increase from -60 to 40 mV, which was initiated after a holding potential of -80 mV. hERG activation curves were fitted by Boltzmann sigmoidal after standardization of tail currents. Cells were evoked through a 2.5-s repolarizing step to +40 mV to inactivate, following a 10 mV stepwise increase from -120 to +20 mV, hERG steady-state inactivation curves were then fitted by Boltzmann sigmoidal. Cells were evoked through a 2.5-s repolarizing step to +40 mV to inactivate, following a 15-s depolarization step. The onset of inactivation curves were recorded with a 10 mV stepwise increase from -120 to +30 mV. Cells were evoked through a 2.5-s repolarizing step to +40 mV to inactivate, following a 15-s depolarization step. The recovery from inactivation curves were recorded with a 10 mV stepwise increase from -120 to +30 mV.

### Western blot

Western Blot analysis was performed as previously described [17]. Briefly, cells were placed on ice and washed thrice with 3 mL ice-cold phosphate-buffered saline (PBS). Then, 60 μL radioimmunoprecipitation assay (RIPA) buffer (Beyotime, Shanghai, PRC) and 0.6 μL phenylmethanesulfonyl fluoride (PMSF, 100 mM, Beyotime) were added to the plates; the cells were then scraped from the plates and transferred into tubes. After three ultrasonic oscillations, the cell lysates were centrifuged at 13,500 rpm for 15 min, and the supernatants were then collected.

The Bradford method was used to determine the total protein concentration. After adding the loading buffer (Beyotime), the samples were boiled and separated using sodium dodecyl sulfate-polyacrylamide gel electrophoresis (SDS-PAGE). Then, the proteins were transferred onto a polyvinylidene fluoride (PVDF) membrane, which was incubated with 5% nonfat milk (Becton Dickinson Co.) for 2 h at room temperature, and then probed with antibodies against hERG (Santa Cruz, USA), Sp1 (Santa Cruz, USA), calnexin (Abcam, ab22683), calreticulin (Abcam, ab22595), Hsp70 (Santa Cruz, USA), Hsp90 (Santa Cruz, USA), ATF-6 (Abcam, ab122897) and actin (Santa Cruz, USA) overnight at 4°C on a shaker. The membranes were washed thrice with 0.05% PBS plus Tween (PBST) and incubated with secondary antibodies (Molecular Probes) for 1 h in the dark at room temperature. The membranes were washed thrice with 0.05% PBST, and then the bands were detected with the Odyssey instrument (American Gene Corp.), finally the Scion Image was used to analyze and quantify the blots.

### Transient transfection

The hERG cDNA with mutations at Y652A (tyrosine to alanine) and F656V (phenylalanine to valine) were generously provided by Professor Guirong Li of Hong Kong University and were transfected into HEK293 cells. The cells were seeded in a 25-mm dish at the desired cell density and incubated for one night. Then, approximately 3–4 h before the transient transfection, we replaced the medium with 4.5 mL DMEM and 250 μL opti-MEM (Gibco) and 16 μL X-treme (Roche) were added to a tube labeled A while 250 μL opti-DMEM and 4 μg cDNA were placed in tube B. Then, the media inside tubes A and B were mixed at room temperature for 20 min, and the mixture was added to the dish containing the cells. The cells were then cultured at 37°C for 48 h before the analysis.

### Immunoprecipitation

The hERG-HEK293 cells were washed thrice with PBS on ice, transferred to a 1.5-mL tube, centrifuged at 3000 rpm for 5 min, and then 300 μL RIPA plus 3 μL PMSF were added to the pellet. The uniformly mixed suspension was placed on ice for 10 min and centrifuged for 10 min at 12,000 rpm. The Bradford method was used to measure the protein concentration of the supernatant. Then, 2 μg each of anti-hERG, Hsp70, or Hsp90 antibody was added and mixed with the protein sample, followed by mixing on a 360° shaker overnight at 4°C. Next, 40 μL of beads (Santa Cruz A-G SC-2003) was added to the mixture, and the tubes were placed on a shaker overnight at 4°C. The mixture was centrifuged at 1,500 rpm for 5 min, and the pellet was washed six times with PBST on ice. In the final step, 100 μL loading buffer was added to the pellets in the tubes, which were boiled for 10 min and then centrifuged. The supernatants were collected, the proteins were separated using SDS-PAGE, and then transferred onto a PVDF membrane. The membrane was blocked with 5% nonfat milk, incubated with anti-hERG, anti-Hsp90, or anti-Hsp70 antibody, followed by the secondary antibody. The Scion Image program was used to analyze and quantify the blots.

### Statistical analysis

The data were expressed at the mean ± standard error of the mean (SEM). An unpaired two-tailed Student’s *t*-test was used to determine the differences among the means, and p-values < 0.05 were considered significant. All graphs were drawn using the GraphPad Prism 5.0 software. The IC_50_ curve was fitted with the Hill equation: Y = bottom + (top - bottom) / (1 + 10 ^ ((logIC_50_ - X) × Hillslope). X: the log dose or concentration. Y: normalized response, top: the maximal response; bottom: the maximally inhibited response. Boltzmann distribution was used to fit the voltage-dependent activation and inactivation curves, and a single exponential function was used to fit the curve of recovery from inactivation (reactivation).

## Results

### DHB immediate perfusion inhibits hERG current concentration-dependently

**Fig 1:**
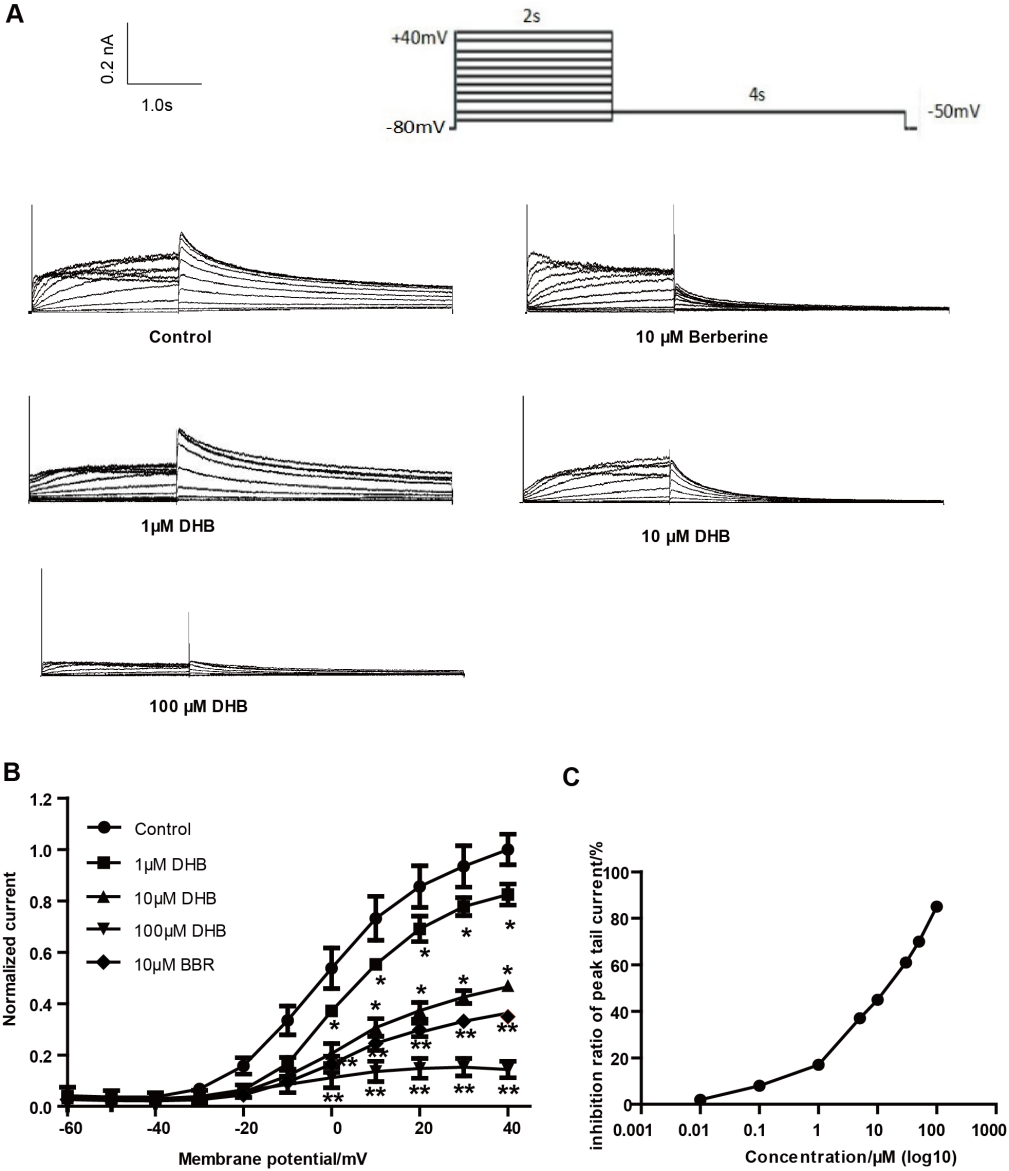
Inhibitive effect of immediate perfusion of DHB on hERG currents. (A) Examples of current traces in control and DHB-treated groups were recorded using illustrated voltage clamp protocol. (B) Normalized I–V relationships for tail current with and without various concentrations of DHB (1, 10 and 100 μM). DHB significantly decreased hERG current. *P < 0.05, **P < 0.01 vs. control, n = 10. (C) Half-maximal inhibitory concentration (IC50) of DHB against hERG current (n = 10).

We determined the transient effect of DHB on hERG current by using the patch-clamp technique. Fig 1 illustrates that hERG tail current concentration-dependently decreased after immediate perfusion with DHB. hERG currents were evoked through a 4-s repolarizing step to -50 mV, following a 2-s depolarization step with a 10 mV stepwise increase from -60 to 40 mV, which was initiated after a holding potential of -80 mV (Fig 1A). Currents were first measured under control conditions (1% DMSO), and then the cells were perfused with varying doses of DHB (1, 10, and 100 μM) for 10 min. Fig 1B depicts the I-V curves of hERG currents acquired in the control and DHB groups. At 40 mV, the inhibition rates in the 1, 10, and 100 μM-treated groups were 17.61% ± 1.52%, 53.44% ± 3.82%, and 85.66% ± 2.77%, respectively. The IC_50_ of DHB against the hERG current was 10.50 μM (Fig 1C) with an nH value of 0.6435.

### DHB accelerates hERG channel inactivation

**Fig 2:**
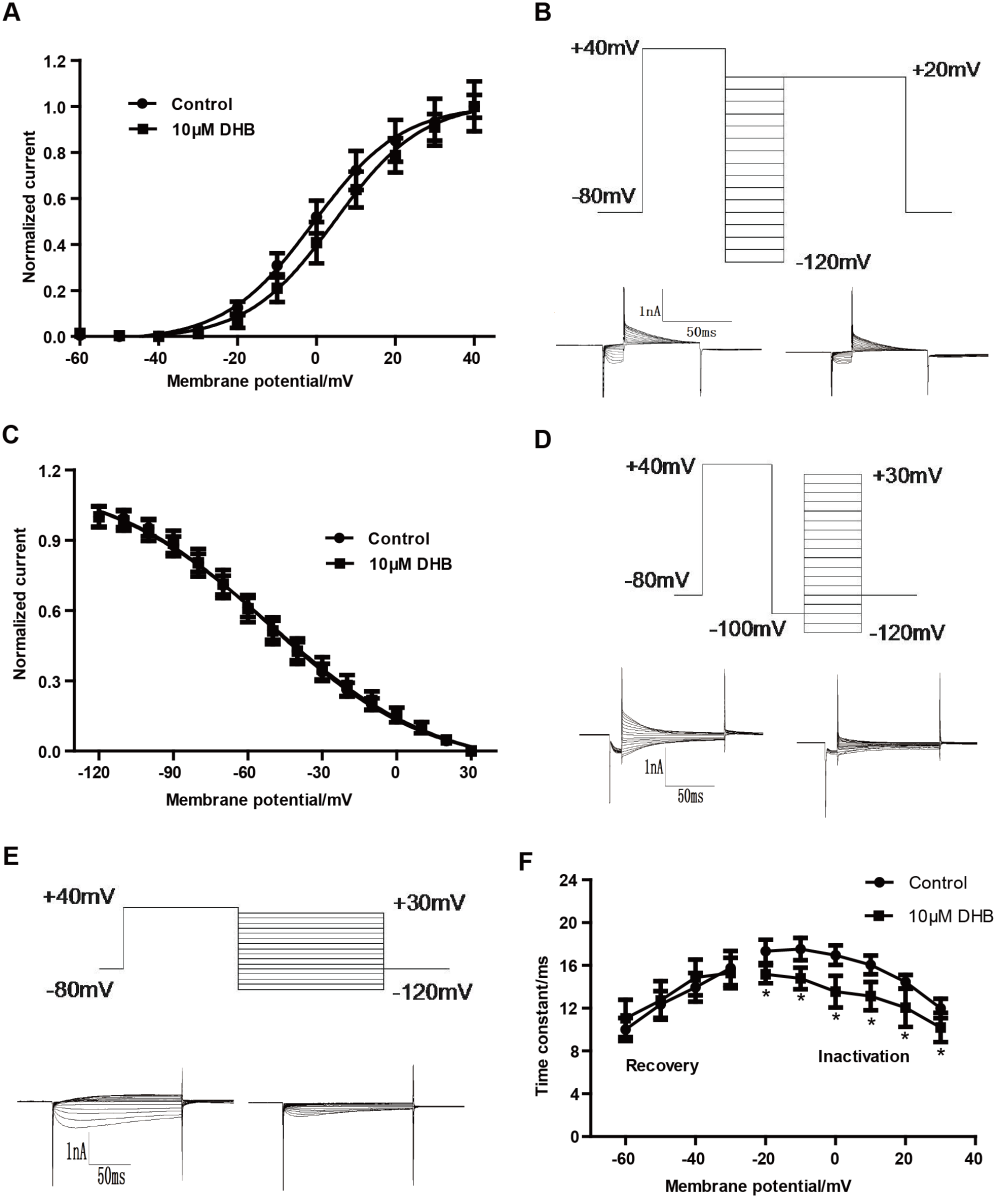
Immediate effects of DHB on hERG channel kinetics. (A) Voltage-dependent activation curves for control and 10 μM DHB-treated group, curves were best fit to a Boltzmann function. (B) Representative current tracing of steady-state inactivation (left and right, for control and 10 μM DHB-treated groups, respectively) using a two-pulse protocol. (C) Normalized steady-state inactivation curves of the control and following exposure to 10 μM DHB. (D) Voltage clamp protocol and representative current tracing for onset of hERG channel inactivation. (E) Voltage clamp protocol and representative current tracing for the recovery from inactivation. (F) Time constant for onset of inactivation and recovery from inactivation before and after exposure to 10 μM DHB. Smooth curves were best fitted of the data to a Boltzmann function. *P < 0.05 vs. control, n = 10.

The voltage-dependent properties or kinetics of ion channel gating are often altered by agents that block ion channels. Therefore, the activation, steady-state inactivation, and the time courses of the hERG channel were examined in the control and the DHB-treated groups in the electrophysiological experiments. The voltage-dependent activation curves shown in Fig 2A were obtained from the tail currents of different test potentials which were normalized and plotted against the test pulse voltages. The Boltzmann function was used to fit the data. As shown in Fig 2A, the half-maximal activation voltage (V_1/2_) values were 0.82 ± 0.75 and 4.68 ± 0.97 mV in the control and 10 μM DHB-treated group, respectively. The corresponding slope factor (*k*) values were 11.23 ± 0.71 and 11.08 ± 0.87, respectively.

Furthermore, we also examined whether DHB inactivated hERG channel. Fig 2B presents the voltage clamp protocol and examples of hERG current traces of steady-state inactivation. Fig 2C shows that immediate perfusion with DHB did not significantly alter the steady-state inactivation curve and the V_1/2_ was -51.59 ± 1.51 and -51.15 ± 1.96 mV for the control and 10 μM DHB-treated groups, respectively, and the corresponding *k* values were -31.90 ± 2.02 and -33.88 ± 2.76, respectively.

We also determined the time courses for the onset of inactivation and recovery from inactivation. The representative traces as well as the illustration of the voltage clamp protocols are shown in Fig 2D and 2E. Furthermore, Fig 2F demonstrates that inactivation of the hERG channel was accelerated after exposure of cells to 10 μM DHB at test potentials between -20 and 30 mV (at -10 mV, τ = 17.53 ± 1.03 and 14.78 ± 1.01 ms in the control and DHB-treated groups). However, the time constant for recovery from inactivation between -60 and -30 mV was not obviously changed by DHB: at -30 mV, τ = 15.74 ± 1.57 and 15.28 ± 1.74 ms in the control and 10 μM DHB-exposed group. The results indicated that DHB inhibited the hERG channel by shortening the time constant of the onset of inactivation.

### DHB reduces mature hERG protein level by inhibiting its trafficking

**Fig 3:**
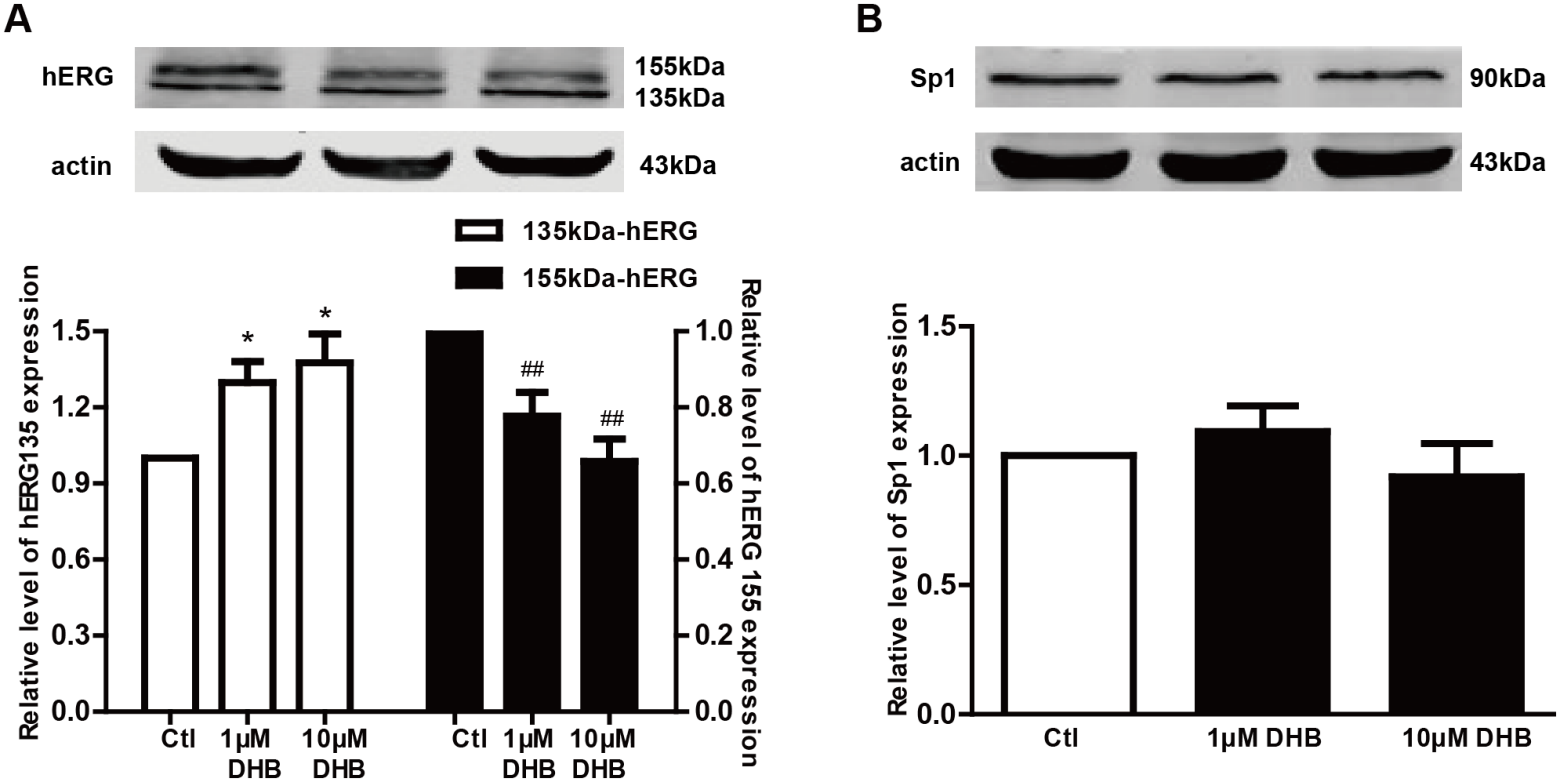
Effects of DHB on the expressions of the hERG protein and transcription facor Sp1 after 24 h incubation. (A) Downregulation and upregulation of 155-kDa and 135-kDa hERG expression, respectively after treatment with different concentrations of DHB (1 μM and 10 μM) for 24 h. *P < 0.05 and ## P< 0.01 vs. control, n = 8. (B) Western blot results of Sp1 expression after 24 h incubation with DHB. The Sp1 expression was unchanged, n = 6.

Western blot was used to demonstrate the effect of DHB on the expresssion of hERG channels. Fig 3A shows that the mature 155-kDa hERG expression decreased after treatment with 1 and 10 μM DHB for 24 h. The 155-kDa band densities of the DHB-treated groups were 77.67% ± 6.25% (1 μM) and 65.8% ± 5.91% (10 μM) of the control level. In contrast, DHB increased the 135-kDa band density by 1.30- (1 μM) and 1.38-fold (10 μM). However, the total amount of hERG protein did not change.

Additionally, Fig 3B illustrates that the expression of the transcription factor Sp1 was not significantly changed after 24 h incubation with DHB. These results suggest that DHB affected the expression of hERG protein by inhibiting hERG channel trafficking rather than affecting transcription.

### DHB treatment decreases hERG current but not hERG channel kinetics

**Fig 4:**
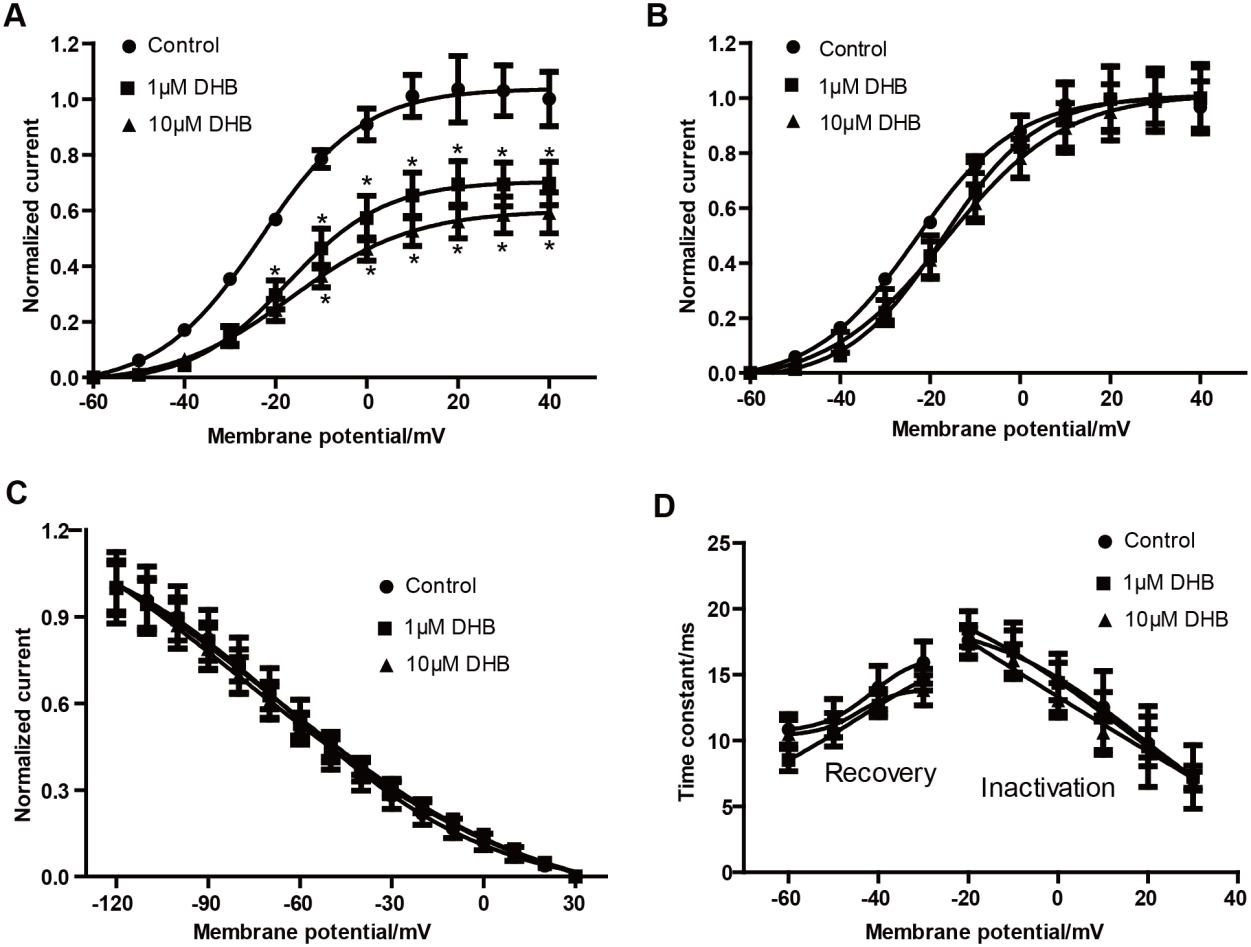
DHB inhibits hERG current but does not affect the hERG channel kinetics after 24 h incubation. (A) Examples of hERG current traces under control and DHB-treated conditions were recorded using the voltage protocol shown in Fig 1A. (B) Voltage-dependent activation curves for the control and DHB-treated groups. (C) Effects of DHB on inactivation curve after 24 h treatment. (D) Effects of DHB on time constant for onset of inactivation and the recovery from inactivation after 24 h treatment. Traces in B-D were recorded using protocols shown in Fig 2. n = 10.

We performed electrophysiological recordings to study the effects of DHB on the hERG current and channel kinetics. As illustrated in Fig 4A, the hERG current significantly decreased in a concentration-dependent manner following DHB treatment (cells incubated with DHB for 24h). Normalized I–V relationships for the tail current before and after exposure to different concentrations of DHB were shown. At 40 mV, the inhibition rates in the 1 and 10 μM DHB-treated groups were 29.26% ± 1.83% and 39.58% ± 2.26%, respectively.

However, DHB did not affect the hERG channel kinetics after 24 h incubation. DHB did not alter the activation curve after incubation for 24 h (Fig 4B) or the steady-state inactivation curve after 24 h incubation (Fig 4C). Furthermore, Fig 4D shows the effect of DHB on the time-constant curve, which did not exhibit any change in the time constant of the onset and recovery from inactivation. Based on these data, we concluded that 24 h incubation with DHB impaired the hERG current in HEK239 cells, but did not affect the hERG channel kinetics.

### DHB acutely blocks hERG channel by binding to aromatic Tyr652 and Phe656 in S6 helix

**Fig 5:**
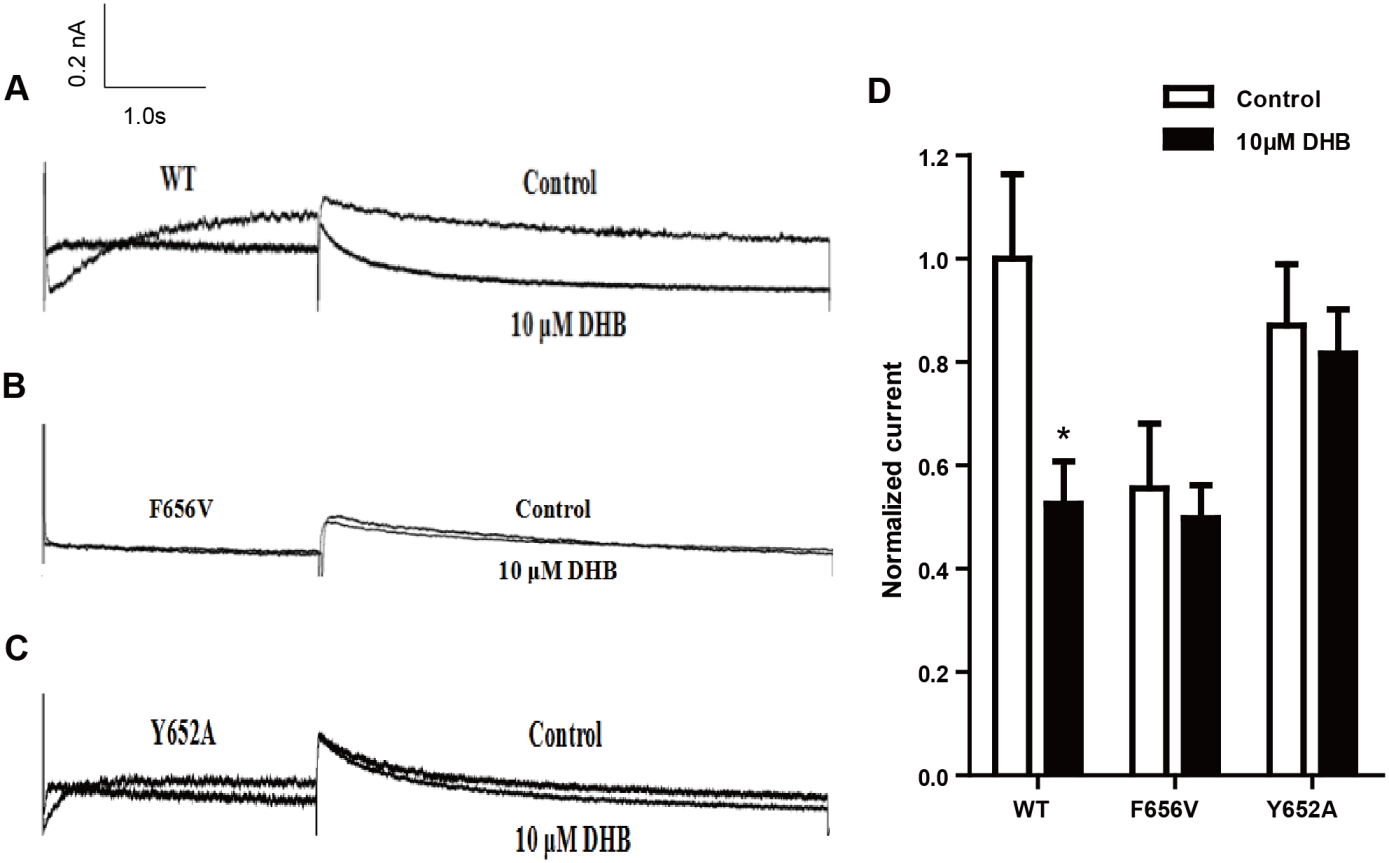
Phe656 and Tyr652 binding mediate acute inhibition of hERG channel. (A-C) Examples of hERG current traces and I–V curves of WT-hERG and mutant hERG current of control and acutely treated DHB groups. (D) Statistical graph of currents. WT-hERG but not Y652A-hERG or F656V-hERG current decreased. *P < 0.05 vs. control, n = 8.

HEK293 cells transiently transfected with hERG cDNA was used to identify the binding sites that induce the acute inhibition of hERG channel triggered by immediate DHB perfusion. The effects of DHB on mutant channels was studied in HEK239 cells transiently transfected with WT-hERG, F656V-hERG or Y652A-hERG. In Fig 5, the WT-hERG tail current significantly decreased after acute perfusion with 10 μM DHB and the inhibition rate at 40 mV was 47.43% ± 4.78%. However, the tail current of Y652A-hERG and F656V-hERG was not affected. The inhibition ratio for F656V-hERG and Y652A-hERG were 10.38% ± 2.52% and 6.25% ± 1.52%, respectively, which was not statistically significant. The results demonstrate that Phe656 and Tyr652 binding accounted for the acute inhibition of the hERG channel.

### Tyr652 binding mediates reduction in hERG expression by DHB

**Fig 6:**
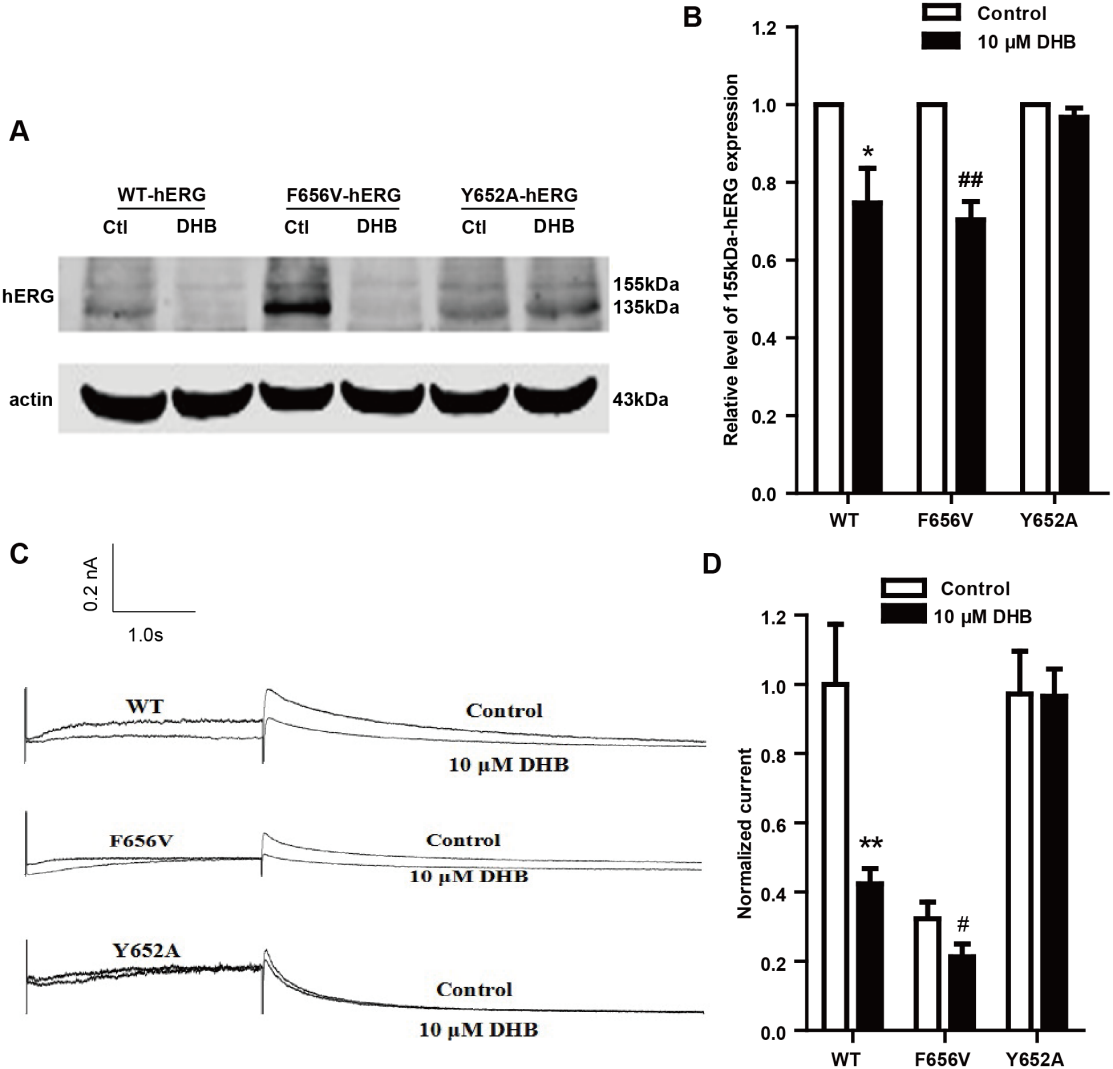
Reduction in hERG expression after 10 μM DHB incubation was mediated by Tyr652 binding. (A) Western blot results and (B) Statistical analysis of WT-hERG and mutant hERG protein of control and DHB-treated groups. Mature WT-hERG and F656V-hERG but not Y652A-hERG decreased after DHB treatment, *P < 0.05, ##*P* < 0.01 vs. control, n = 6. (C) Examples of hERG current traces and I–V curves of WT-hERG and mutant hERG current of control and incubation treated DHB groups. (D) Statistical graph of currents. WT-hERG and F656V-hERG but not Y652A-hERG current decreased. **P < 0.01, #*P* < 0.05 vs. control, n = 6.

To determine the binding sites mediating the reduction in hERG expression after incubation of cells with DHB, we examined the effects of DHB on mutant channels. Fig 6B shows the results of the western blot analysis of hERG expression. The expression of 155-kDa hERG protein in the WT-hERG and F656V-hERG cells remarkably decreased after treatment with 10 μM DHB, with band densities of 74.67% ± 8.91% and 70.34% ± 4.67% compared with the control level. However, DHB showed no remarkable effect on the 155-kDa hERG expression of Y652A-hERG cells. Fig 6D shows the results of the hERG current traces of the WT, F656V and Y652A in the absence and presence of DHB overnight incubation. The WT-hERG tail current significantly decreased after overnight incubation with 10 μM DHB and the inhibition ratio at 40 mV was 57.64% ± 13.27%. The inhibition ratio for F656V-hERG and Y652A-hERG were 40.27% ± 7.67% and 5.67% ± 1.19%, respectively. 10 μM DHB showed no remarkable inhibitory effects on Y652A-hERG currents. These results suggest that the reduction in hERG expression after DHB incubation was on account of Tyr652 binding.

### Channel folding impairment results in trafficking deficiency

**Fig 7:**
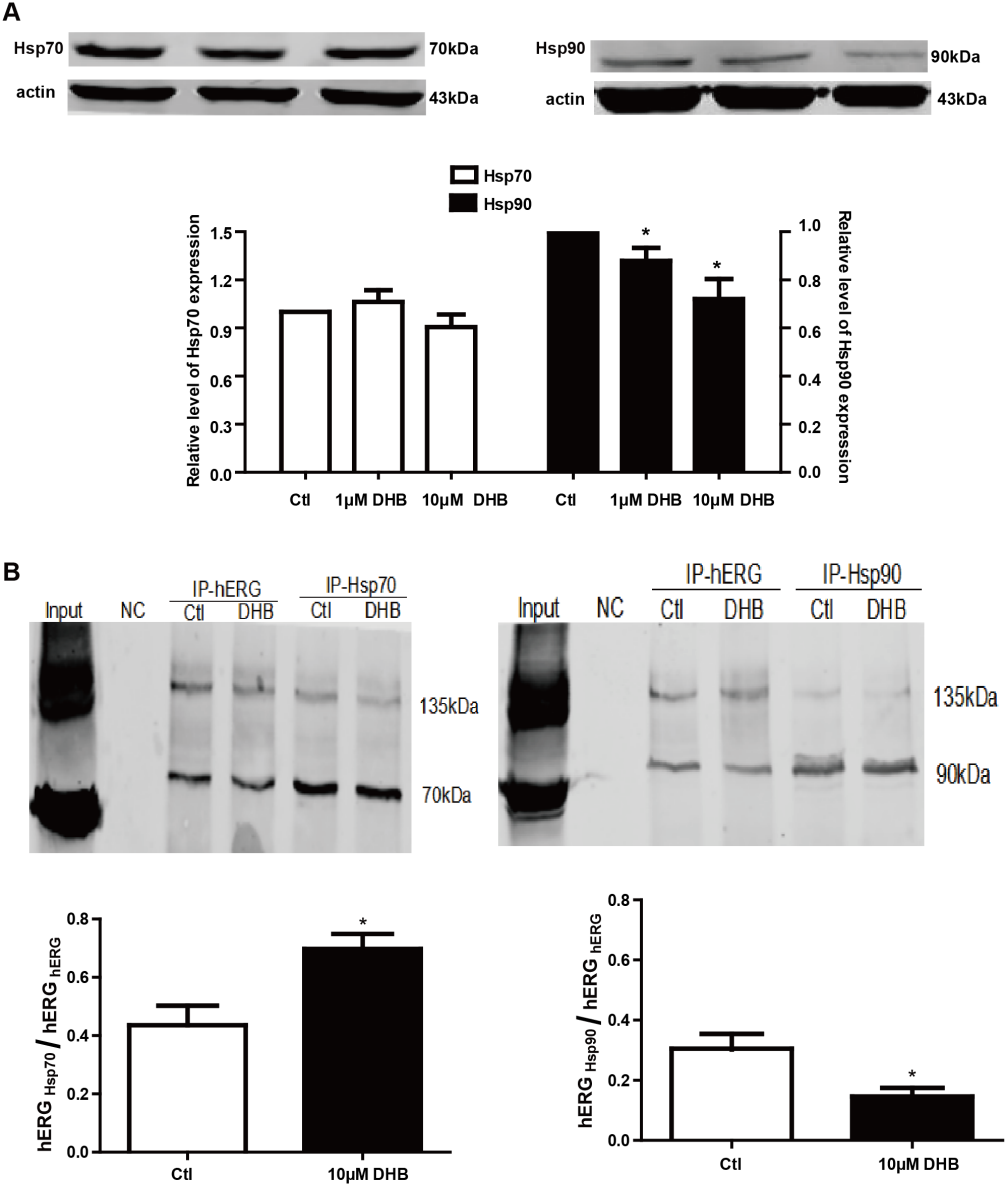
DHB disrupts interactions between hERG and chaperones. (A) Western blot analysis of Hsp70 and Hsp90 after 24 h incubation with 1 μM and 10 μM DHB. Hsp90 expression was concentration-dependently reduced while Hsp70 expression did not change. *P < 0.05 vs. control, n = 4. (B) Immunoprecipitation results show increased and decreased interaction between hERG and Hsp70 or Hsp90 respectively after treatment with 10 μM DHB. *P < 0.05 vs. control, n = 3.

Channel folding is considered a critical element in channel trafficking. Therefore, western blot and immunoprecipitation were used to detect the folding of hERG channels to elucidate the mechanism of the DHB-induced trafficking deficiency. The chaperones Hsp70 and Hsp90 participate in channel folding; hence, we examined their expression after DHB incubation for 24 h. Fig 7A illustrates that the expression of Hsp90 but not Hsp70 reduced by 12.08% ± 5.34% and 27.99% ± 8.33% after incubation with 1 and 10 μM DHB, respectively. Fig 7B shows that the interaction between hERG protein and Hsp90 remarkably reduced, whereas the interaction between hERG protein and Hsp70 increased.

### *DHB causes unfolded protein response* (UPR)

**Fig 8:**
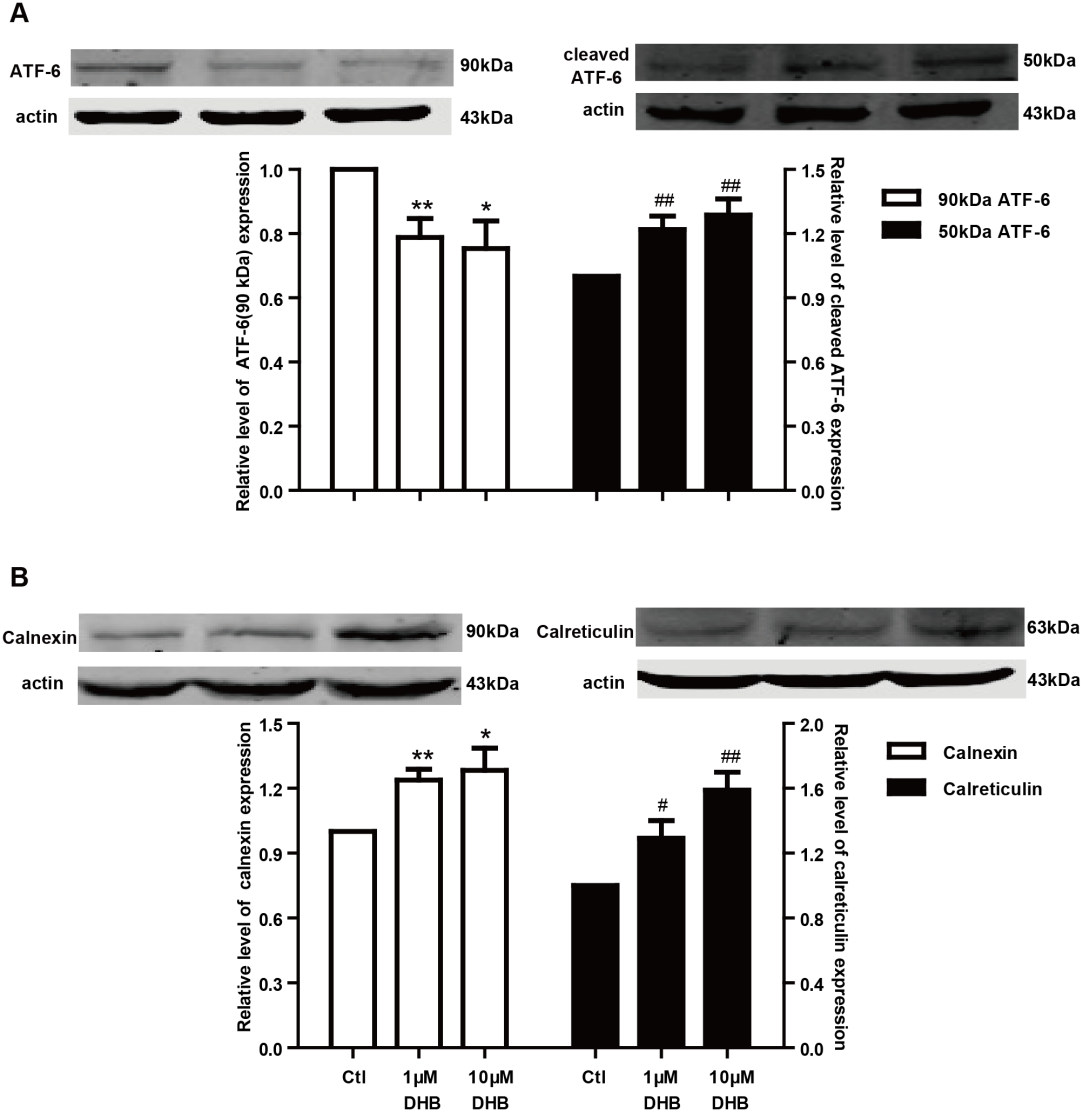
Unfolded protein response is activated by DHB. (A) DHB incubation for 24 h significantly decreased ATF-6 (~90 kDa) expression and proportionally increased cleaved ATF-6 (~50 kDa) expression. *P < 0.05 and #*P* < 0.05 vs. control, n = 6. (B) Western blot analysis shows DHB markedly increased the expression of both chaperones, calnexin and calreticulin. *P < 0.05, **P < 0.01, #*P* < 0.05, and ##*P* < 0.01 vs. control, n = 6.

The UPR plays a vital role in the prevention of unfolded protein accumulation in the ER. Therefore, we studied the UPR to determine the effects of channel folding impairment. Cleaved ATF-6 (~50 kDa) is a marker protein of the UPR, which is generated from ATF-6 (~90 kDa). Fig 8A illustrates that ATF-6 expression significantly reduced while that of cleaved ATF-6 proportionally increased. These findings suggest that the UPR was activated after incubation with DHB for 24 h. Calnexin and calreticulin are chaperone proteins, which assist in the folding of hERG proteins and are the downstream targets of cleaved ATF-6. Based on this, the expression of calnexin and calreticulin was examined after DHB incubation. As depicted in Fig 8B, the expression of calnexin and calreticulin significantly increased after incubation with DHB for 24 h.

### DHB increased degradation of mature hERG channel

**Fig 9:**
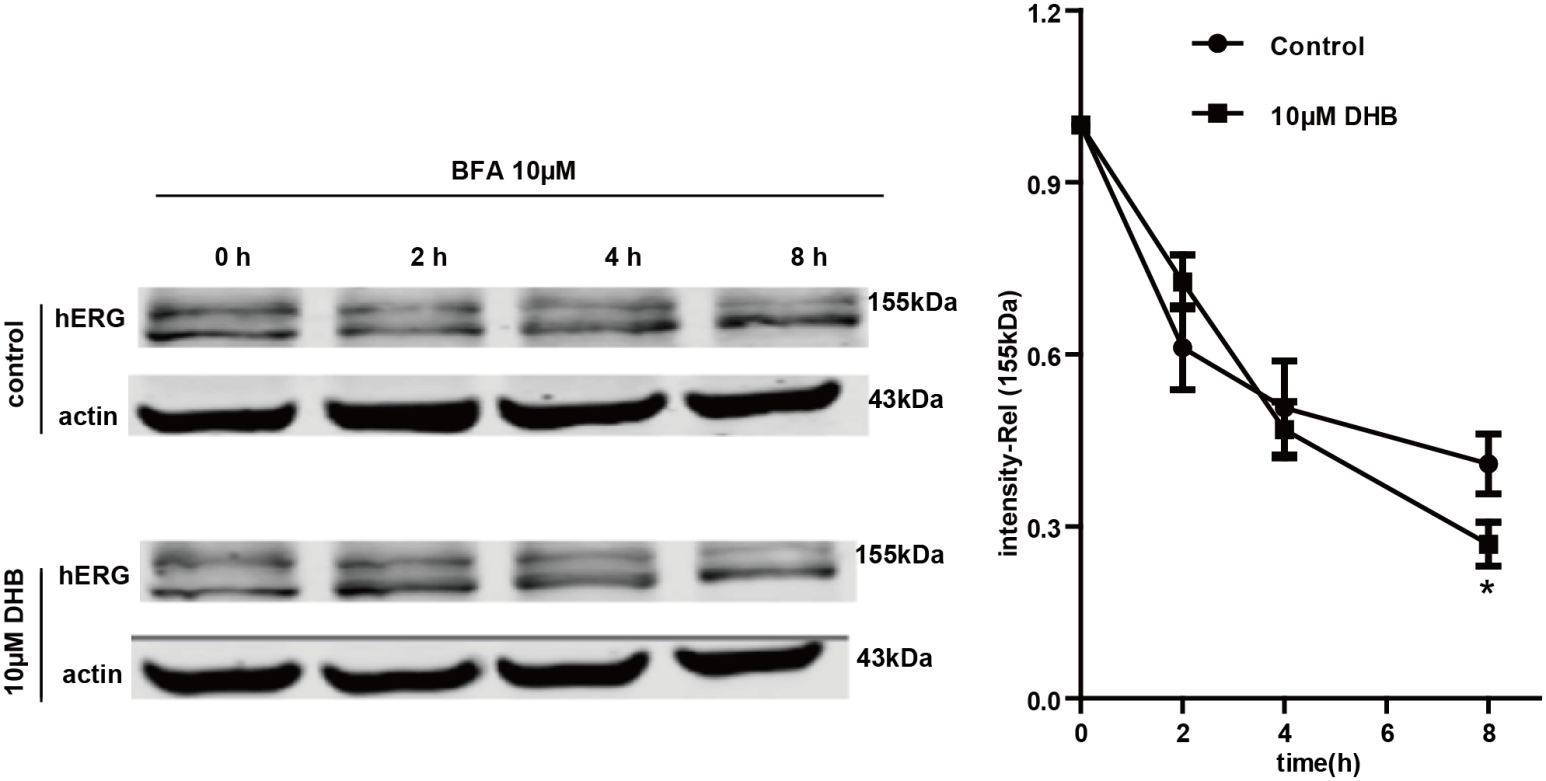
Western blot analysis suggests that DHB accelerated mature 155-kDa hERG degradation. hERG-HEK cells were treated with or without 10 μM DHB in the presence of BFA, which inhibits hERG protein transportation from the ER to the Golgi body. Cells were harvested and assayed using western blot at the designated time points. Intensities of the mature 155-kDa hERG bands were normalized to the time 0 h value. *P < 0.05 vs. control, n = 4.

The Golgi transit inhibitor brefeldin A (BFA) exerts an inhibitory effect on the conversion of the immature 135-kDa hERG to the mature 155-kDa hERG [18]. Therefore, we used BFA to determine the effects of DHB on the degradation of the mature hERG channel. The hERG-HEK cells were first treated with 10 μM BFA for 1 h and then cultured in the absence or presence of 10 μM DHB in the presence of BFA. Western blot was subsequently used to examine the 155-kDa hERG expression at various time points after BFA treatment. The degradation rate of the mature 155-kDa hERG was demonstrated by the normalized 155-kDa hERG band intensity. As illustrated in Fig 9, the 155-kDa hERG expression decreased time-dependently in the presence of BFA. The 155-kDa band intensity decreased by approximately 45% in control cells after incubation with BFA for 8 h; however, treatment with DHB exhibited a reduction of approximately 65%. These results demonstrate that the degradation rate of the mature 155-kDa hERG remarkably increased after exposure to 10 μM DHB.

## Discussion

Drug-induced cardiotoxic events are significantly involved in the QT prolongation caused by hERG channel deficiency [19]. This deficiency usually results from direct blockade of the hERG channel or the indirect impairment of hERG protein synthesis, trafficking, or degradation [20]. In our study, DHB inhibited the hERG current in a concentration-dependent manner after immediate perfusion, and the IC_50_ was 10.50 μM. DHB also affected hERG channel kinetics by accelerating the inactivation of the channel and eventually reducing the instantaneous inactivation time constant of the hERG channel, which was caused by direct disruption of the hERG channel gating. In addition, we detected the binding determinant of this direct blockade. Numerous studies have shown that agents can interact with the aromatic residues of the helixes of hERG channels, thereby exerting their effects [21]. The aromatic amino acid residues Tyr652 and Phe656 in S6 helix are regarded as crucial molecular determinants of hERG channel blockade by agents [22]. In this study, we found that the tail current of Y652A-hERG or F656V-hERG did not significantly decrease by immediate perfusion with DHB, indicating that Phe656 and Tyr652 binding account for the direct impairment of hERG channel. Thus, in the present study, we aimed to deternmine the underlying mechanisms involved in the process of DHB treatment. In short, our date demonstrated that DHB acutely blocked hERG channels and poteintially decreased hERG plasma membrane expression through two pathways involving disruption of forward trafficking of immature hERG channels and enhanced degradation of mature hERG channels.

Althrough previous study has already demonstrated that DHB was the most potent hERG inhibitor [23], the underling mechanism is still uncertain. In the current study, we first demonstrated that DHB acutely blocked hERG channel by binding to the aromatic Tyr652 and Phe656. DHB decreases hERG plasma membrane expression through two pathways involving the disruption of the forward trafficking of immature hERG channels and the enhancement of the degradation of the mature hERG channels. Our findings suggested that the forward trafficking disruption was caused by the impairment of channel folding, which was associated with the changes in the interactions between hERG proteins and chaperones. We speculated that DHB was bound to the hERG channels and caused a conformational change that led to abnormal trafficking. This effect impaired the transportation of the channel to the cell membrane, and the binding determinant was Tyr652. Furthermore, trafficking inhibition activated the UPR, and degradation of the mature hERG channels was increased by DHB.

It is well known that the two glycosylation processes are indispensable for maturation of the hERG channel. After synthesis and initial glycosylation in the ER, the immature 135-kDa hERG is trafficked to the Golgi body where it is further glycosylated into the mature 155-kDa hERG [24]. Our data showed that the mature 155- kDa hERG decreased while the immature 135-kDa hERG increased after DHB incubation for 24 h, while the expression of the transcription factor Sp1 remained unchanged. Our data indicated that the forward trafficking from the ER to the Golgi of hERG channel was disrupted. Notably, deficient protein trafficking, biogenesis disturbance, is an emerging mechanism for drug-induced hERG channel defects [25, 26]. Studies have shown that the defective trafficking of hERG channels commonly results from the misfolding of hERG proteins. Chaperones bind to channel proteins and, thereby, assist in proper folding, and the molecular chaperones Hsp70 and Hsp90 perform vital functions in channel folding [27, 28]. Hsp70 interacts with the nascent hERG in the ER while Hsp90 interacts with the terminal hERG in the cytoplasm to facilitate folding [29]. In this study, 1 and 10 μM DHB incubation decreased the expression of Hsp90, whereas did not affect Hsp70 expression. The interaction between hERG protein and Hsp90 reduced while the interaction between hERG protein and Hsp70 increased by DHB. Hsp90 was downregulated by DHB, which may have directly decreased the interaction between the terminal hERG protein and Hsp90 and resulted in an accumulation of a large quantity of hERG protein that folded incorrectly. This effect may have increased the interaction between the initial hERG and Hsp70. Another possible reason for this is that DHB may change the conformation of the hERG channel. Therefore, it would be difficult for Hsp70 to recognize and interact with the hERG protein, thereby elongating the interaction of the early hERG protein with Hsp70 and reducing the interaction of hERG protein with Hsp90. These findings suggested that DHB-induced hERG trafficking defect results from protein misfolding, which may due to the reduction of Hsp90 expression and the aberrant interaction between hERG protein and molecular chaperones. Moreover, we determined the binding sites responsible for mediating the reduction in hERG expression after DHB incubation. We found that the 155-kDa hERG protein expressions of the WT-hERG and F656V-hERG remarkably decreased by 10 μM DHB. However, DHB showed no remarkable effect on the 155-kDa hERG expression of Y652A-hERG. Statistical analysis of the hERG current in the absence and presence of DHB overnight incubation showed similar results to those of the protein expression analysis. These results suggested that DHB binds to the hERG channels causing a conformational change, which leads to abnormal trafficking. Therefore, transport of the channel to the cell membrane is disrupted, and the binding determinant is Tyr652. We also detected the effects of DHB on the hERG current and channel kinetics. The hERG current significantly decreased in a concentration-dependent manner, but DHB did not affect the hERG channel kinetics after 24 h incubation. This was likely attributable to the enhanced degradation of the mature form of the hERG channel described below.

The accumulation of the misfolded or imperfectly folded hERG proteins could cause UPR, which is an endoplasmic reticulum stress response that promotes the generation of the chaperones and serves a vital role in the prevention of unfolded proteins accumulation [30]. The UPR is also implicated in the promotion of the degradation pathway [25]. ATF-6, inositol-requiring enzyme 1 (IRE-1), and protein kinase R (PKR)-like endoplasmic reticulum kinase (PERK) are the three pathways of UPR, and ATF-6 is considered a major factor in the initiating stage of the response [31]. The cleaved ATF-6 (~50 kDa) is a marker protein of the UPR, which is generated from ATF-6 (~90 kDa). The cleaved ATF-6 can shuttle into the nucleus and induce the transcription of UPR-associated genes, which enhances the expression of the chaperones, thereby facilitating protein folding [32]. Calnexin and calreticulin are crucial chaperones that serve as target proteins of the cleaved ATF-6 and aid in channel folding [33].

Our findings suggested that DHB obstructed hERG channel trafficking. Consistent with this observation, we found that the ATF-6 (~90 kDa) expression significantly reduced while that of cleaved ATF-6 (~50 kDa) proportionally increased after incubation with DHB. Furthermore, the expressions of calnexin and calreticulin markedly increased after 24 h incubation with DHB. These findings demonstrated that the hERG trafficking defect induced by DHB activates UPR. Additionally, ER-associated degradation can degrade the misfolded and trafficking-deficient proteins retained in the ER [15, 34]. Moreover, we inferred that the same agent not only disrupted hERG trafficking but also increased the degradation of the mature hERG protein [15]. Furthermore, the western blot results revealed that the degradation rate of the mature 155-kDa hERG was enhanced after exposure to 10 μM DHB.

Althrough the studies about hERG channel are an important part of cardiac safety pharmacology, certain limitations to our study should be considered. In the present study, we do not take into consideration the multi ion channel block effects of DHB, which has been presented by a recent review on hERG channel [35]. This updated and comprehensive cardiac risk assessment strategy can help us to get a better understanding of the undrlying mechanisms during the blockage of ion channels including hERG channel by small molecules. In the future studies, we will focus on more ion channels which could be potentially affected by the compound of interest. Additionally, the absorption and metabolism of drugs in vivo are extremely complicated, it is not persuasive enough to simply described the pharmacokinetic results of 10 μM DHB on the hERG channel, various factors such as bioavailability, plasma free concentration, and intracellular levels of DHB should be taken into account. Due to the lack of in vivo trials to determine the relationship between hERG inhibition and the possible drug-induced LQTS, more animals and clinical trials are needed in the further studies.

In summary, we demonstrated that DHB directly blocked the hERG channel by binding to the aromatic Tyr652 and Phe656. Further, we suggested that DHB decreased hERG plasma membrane expression through two pathways: the disruption of the forward trafficking of immature hERG channels and the enhancement of the degradation of the mature hERG channels. The forward trafficking disruption was caused by impaired channel folding, which was associated with changes in the interactions between hERG proteins and chaperones. Further, the trafficking inhibition activated the UPR, and the degradation of the mature hERG channels was enhanced by DHB. Together, our findings shed light on the potential mechanisms of the DHB-induced hERG channel deficiency which may provide a better understanding of drug-induced LQTS. The results also provide valuable information and theoretical basis for development and adverse reactions prevention of DHB.

## Supporting information

S1 Fig Supplemental results and statistical date for Figs 1–9. (RAR)
